# Depression and cardiovascular disease are not linked by high blood pressure: findings from the SAPALDIA cohort

**DOI:** 10.1038/s41598-022-09396-2

**Published:** 2022-04-01

**Authors:** Katrina A. Obas, Marek Kwiatkowski, Emmanuel Schaffner, Undine E. Lang, Daiana Stolz, Ikenna C. Eze, Medea Imboden, Nicole Probst-Hensch

**Affiliations:** 1 Department of Epidemiology and Public Health, Swiss Tropical and Public Health Institute, Kreuzstrasse 2, 4123 Allschwil, Switzerland; 2 University of Basel, Basel, Switzerland; 3 University Psychiatric Clinics, Basel, Switzerland; 4 Clinic of Respiratory Medicine and Pulmonary Cell Research, University of Basel, Basel, Switzerland

**Keywords:** Hypertension, Psychology, Cardiology, Public health, Epidemiology, Epidemiology, Risk factors

## Abstract

Depression and cardiovascular disease (CVD) are main contributors to the global disease burden and are linked. Pathophysiological pathways through increased blood pressure (BP) are a common focus in studies aiming to explain the relationship. However, studies to date have not differentiated between the predictive effect of depression on the course of BP versus hypertension diagnosis. Hence, we aimed to elucidate this relationship by incorporating these novel aspects in the context of a cohort study. We included initially normotensive participants (n = 3214) from the second (2001–2003), third (2009–2011), and fourth (2016–2018) waves of the Swiss Cohort Study on Air Pollution and Lung and Heart Diseases in Adults (SAPALDIA). We defined depression based on physician diagnosis, depression treatment and/or SF-36 Mental Health score < 50. The prospective association between depression and BP change was quantified using multivariable censored regression models, and logistic regression for the association between depression and incident hypertension diagnosis. All models used clustered robust standard errors to account for repeat measurements. The age-related increase in systolic BP was slightly lower among people with depression at baseline (β = − 2.08 mmHg/10 years, 95% CI − 4.09 to − 0.07) compared to non-depressed. A similar trend was observed with diastolic BP (β = − 0.88 mmHg/10 years, 95% CI − 2.15 to 0.39), albeit weaker and not statistically significant. Depression predicted the incidence of hypertension diagnosis (OR 1.86, 95% CI 1.33 to 2.60). Our findings do not support the hypothesis that depression leads to CVD by increasing BP. Future research on the role of depression in the pathway to hypertension and CVD is warranted in larger cohorts, taking into account healthcare utilization as well as medication for depression and hypertension.

## Introduction

Depression is a leading cause of disability worldwide affecting more than 264 million people^1^. Depression is also an independent risk factor for cardiovascular disease (CVD)^2,3^, which continues to cause the greatest disease burden worldwide^4^. The mechanisms underlying the relationship between depression and CVD are not yet elucidated. Pathophysiological pathways involving increased blood pressure (BP) have been the focus of proposed explanations linking depression and CVD^5–8^. Given the heavy burden these conditions cause globally, it is of great public health relevance to investigate the potential causal role of depression in the course of BP to provide greater insight into the potential of depression control in reducing CVD risk and the global disease burden.

Several studies propose mechanisms mediating the relationship between depression and CVD^5–8^, and focus on shared pathways with high BP. Firstly, depression and BP are linked by lifestyle. Depression is associated with an increased risk for unhealthy behaviours such as smoking, physical inactivity, increased alcohol consumption, poor nutrition, and poor sleep^9–11^, all of which are known risk factors for raised BP. Secondly, biological mechanisms such as autonomic nervous system dysfunction are associated with depression, resulting in decreased heart rate variability and increased heart rate^12^, which in turn lead to an increase in BP. Thirdly, there is evidence that antidepressant treatments themselves may independently increase BP^13,14^.

The evidence on the prospective associations between depression and BP is currently mixed. Longitudinal studies have concentrated on the predictive association between depression and incident hypertension^13,15–17^. A meta-analysis^18^ of 9 prospective studies revealed that depression increased the risk of incident hypertension (RR 1.42, 95% CI 1.09 to 1.86). Although some studies detected hypertension with BP measurements alone^19,20^ or in combination with hypertension diagnosis or antihypertensive medication use^13,15,17,21–24^, several others relied solely on physician-diagnosed hypertension and the use of antihypertension medication to assess the presence of hypertension^16,25–27^. However, many people remain unaware that they have high BP, especially if they do not experience symptoms and fail to get a diagnosis. Depression is associated with higher healthcare utilization^28^, thus people living with depression might be more likely to have underlying hypertension diagnosed than those without depression.

Taking direct BP measurement into consideration is important in studies on depression and hypertension. Yet, few longitudinal studies have assessed the association between depression and BP as a continuous variable. One population-based study^29^ and a study among people with hypertension^30^ provided evidence that baseline depression predicted lower BP^29,30^. Other population-based studies reported that BP increased with increasing^31^ or consistently high^17^ depressive symptoms. In summary, the little evidence that exists on the effect of depression on BP is mixed. A deeper understanding of how depression affects BP among normotensive people would be particularly valuable from a prevention perspective.

**Figure 1:**
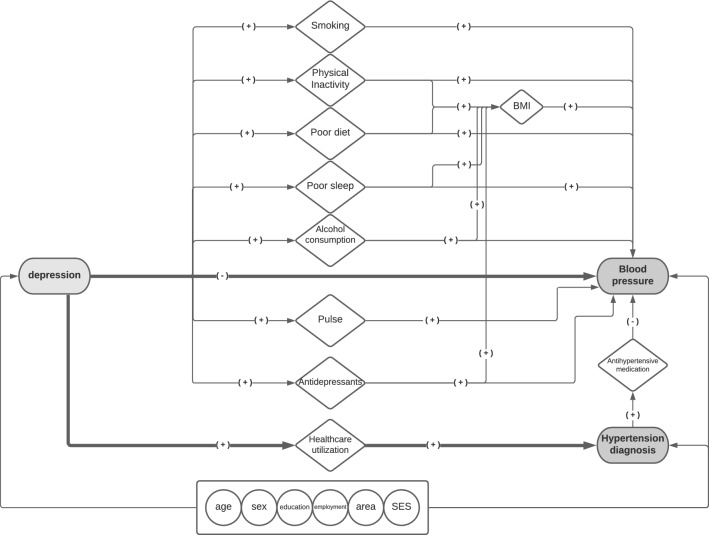
Conceptual Framework. The figure depicts the conceptual framework of the study’s main associations of interest (bold lines) between depression and (**a**) change in blood pressure and (**b**) incident hypertension diagnosis, including the confounders (circles) and mediators (rhombus), based on prior knowledge. A (+) indicates a positive association and (−) indicates a negative association. SES: socioeconomic status. BMI: body mass index.

The goal of this study was to elucidate the relationship between depression and the course of BP among normotensive people. We therefore assessed separately the prospective association between depression and the following BP-related outcomes: (a) change in systolic and diastolic BP and, (b) incident hypertension diagnosis. Figure 1 depicts the hypothesized relationships between depression, BP and hypertension based on prior knowledge.

## Methods

### Participants

**Figure 2:**
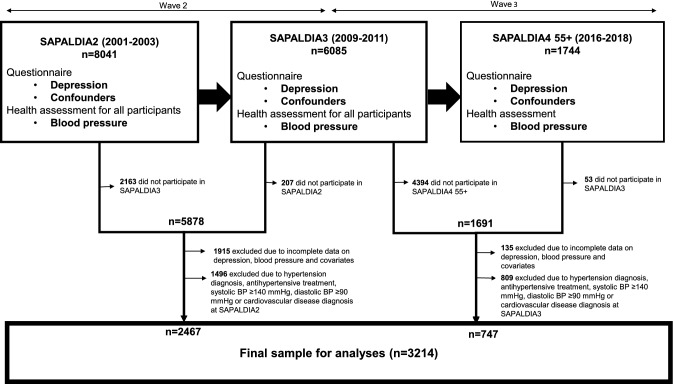
Flow diagram for the inclusion of study participants. The figure summarizes the sources of variables included in the present study as well as the flow diagram for the inclusion of study participants in the repeat measurements analyses. Participants from SAPALDIA2, SAPALDIA3 and SAPALDIA4 55+ were included in the study if within a wave (either SAPALDIA2 to SAPALDIA3 or SAPALDIA3 to SAPALDIA4 55+) they had complete data on systolic and diastolic blood pressure at baseline and follow-up, complete baseline data on depression, potential mediators and confounders, and excluded if at baseline they reported a physician diagnosis of hypertension or cardiovascular disease, antihypertension treatment, or measured high blood pressure (≥ 140/90 mmHg).

We used longitudinal data from the Swiss Cohort Study on Air Pollution and Lung and Heart Diseases in Adults (SAPALDIA) which began in 1991 (SAPALDIA1) with 9651 randomly selected participants aged 18 to 60 from eight representative Swiss areas. SAPALDIA1 focused on air pollution and respiratory health and expanded into cardio-metabolic outcomes and wellbeing thereafter (SAPALDIA2 (2001–2003), SAPALDIA3 (2009–2011), SAPALDIA4 (2016–2018)). The current study used data collected from SAPALDIA2, SAPALDIA3 and SAPALDIA4 for longitudinal analyses. At each of these follow-ups, participants completed a health examination and questionnaire covering their lifestyle and health status including information on physician diagnosis, and medication use for depression and hypertension. While all participants at SAPALDIA2 and SAPALDIA3 were subjected to a health assessment, the health examination in SAPALDIA4 was restricted to participants aged 55 years and older at the time (SAPALDIA4 55+). Details of the SAPALDIA study protocols are provided elsewhere^32–34^. The overall participation rate at SAPALDIA1 (n = 9651) was 59.3% of the sample frame^34^, with a retention rate of 83% from SAPALDIA1 to SAPALDIA2 (n = 8047), 76% from SAPALDIA2 to SAPALDIA3 (n = 6088), and 85% from SAPALDIA3 to SAPALDIA4 (n = 5149). Of the 5149 SAPALDIA4 participants, 2179 were eligible for the 55+ health exam of whom 1746 underwent it^33^. For our longitudinal analyses, we pooled data from two waves, SAPALDIA2 to SAPALDIA3 (wave 2) and SAPALDIA3 to SAPALDIA4 55+ (wave 3). Participants were included if they met the following criteria within a wave: (1) had complete data on systolic and diastolic BP at first (termed: “baseline”) and second time point (termed “follow-up”) of each wave; (2) had complete baseline data on depression, potential confounders and mediators, (3) did not report a physician diagnosis of hypertension or CVD at baseline, (4) had no history of antihypertension treatment at baseline, and (5) were normotensive (systolic BP < 140 mmHg and diastolic BP < 90 mmHg) at baseline (n = 3214). A flow diagram for the inclusion of study participants is depicted in Fig. 2. The number of participants meeting these criteria was n = 2467 for wave 2 only, n = 171 for wave 3 only, and n = 576 for both waves. Data use from wave 1 was impeded due to the lack of BP data at the SAPALDIA1 survey.

Ethical approvals for the SAPALDIA studies were obtained from Ethics committees of North-West Switzerland, and the Swiss Academy of Medical Sciences. SAPALDIA complies with the Declaration of Helsinki. All participants provided informed written consent before participating in any aspect of the SAPALDIA studies.

### Depression

The presence or absence of depression at baseline (first time point of each wave) was deduced from the following information provided by the participants: First, participants responded to questions about having physician-diagnosed depression and provided a medication list from which antidepressant medication use was derived. Second, participants completed the Medical Outcomes Study Short Form 36 questionnaire (version 1)^35^. The Mental Health domain (SF-36 MH) scores ranged from 0 to 100 and a score below 50 was found to be an appropriate cut-off to screen for depressive disorders^36^. We therefore defined depression as self-reported physician-diagnosed depression or a history of antidepressant use (ATC codes starting with N06A) or an SF-36 MH score < 50. We considered also depression disaggregated by any antidepressant use, and by antidepressant class (N06AA—Non-selective monoamine reuptake inhibitors; N06AB—Selective serotonin reuptake inhibitors; N06AX/combination—other antidepressants and/or using a combination of antidepressants) to assess whether the associations of interest were modified by antidepressants.

### Change in blood pressure and incident diagnosis of hypertension

At each health assessment of SAPALDIA2, SAPALDIA3 and SAPALDIA4 55+ surveys, BP was measured twice by trained field workers using an automatic device (705CP and M6, OMRON, Tokyo, Japan) with a cuff of appropriate size (after having measured arm circumference) and using the Riva-Rocci method, in the sitting position after minimum 10 min rest, on the left arm, with 3 min between measurements. The first and second BP measurements were averaged. To obtain the change in BP from SAPALDIA2 to SAPALDIA3 and SAPAPALDIA3 to SAPALDIA4 55+, we subtracted the baseline BP from the follow-up BP within a wave. The coefficients of the censored regression models are interpretable as the difference between depressed and non-depressed participants in age-related increase in BP over 10 years in mmHg. A positive coefficient for the depression variable represented a larger age-related increase in BP among depressed compared to non-depressed people, while a negative coefficient represented a smaller age-related increase in BP among depressed compared to non-depressed people over the wave. Because the length of time between follow-ups varied between 5.6 and 9.7 years among participants, we expressed the observed changes as a per-decade rate, by dividing them by the individual durations of follow-up and multiplying by 10.

Incident hypertension diagnosis was deduced from participants’ data on physician diagnoses and medication intake. Participants were identified as having incident diagnosis of hypertension if they had physician-diagnosed hypertension or used antihypertensive medication, regardless of the BP level at corresponding health examination. Participants with high BP level (> 140/90 mmHg) at examination but who reported neither physician-diagnosed hypertension nor antihypertensive medication intake were considered as not having incident diagnosis of hypertension.

### Covariates

We selected potential confounders for inclusion in our models based on prior knowledge. These included the participants’ age (years), sex (male, female), education (primary school (≤ 9 years), secondary school (> 9– ≤ 12 years), technical college or university (≥ 12 years)), employment status (employed, house person, in training/military service, not working, pensioner), study area (Basel, Wald, Davos, Lugano, Montana, Payerne, Aarau, Geneva), and Swiss socioeconomic position (SSEP) which is a neighborhood socioeconomic status index based on the 2000 census data covering education and occupation of households members as well as room occupancy and rents of households in a neighborhood^37^.

In the analysis of the association between depression and change in BP, we considered sex and antidepressant use as potential effect modifiers. We also considered the following as potential mediators: smoking status (never, former, smoker), moderate to vigorous physical activity as per World Health Organization recommendations (less versus more than 150 min per week)^38^, alcohol consumption (≤ 1/ > 1 glass per day), vegetable and fruit consumption (not daily/daily), daytime sleepiness (mean item score from the 8-item Epworth’s Sleepiness Scale)^39^, body mass index (kilograms/meter^2^), and pulse (beats per minute).

### Statistical analysis

#### Depression and change in blood pressure

Many study participants who were normotensive at baseline developed hypertension and were prescribed antihypertensive medication prior to their follow-up health assessments. Correctly accounting for this treatment is of paramount importance in statistical analyses of BP. Several analytical strategies have been evaluated in a simulation study^40^, which recommended the use of censored normal regression. Our own ad-hoc simulations (not reported) corroborated this assessment. In this approach, the BP change for all participants diagnosed or treated for hypertension during a wave was right-censored at the measured value. This is equivalent to assuming that had these participants not received the diagnosis, their BP at the follow-up assessment would have been equal or greater than the value that was actually measured. The coefficients fitted by censored regression have the same familiar interpretation as those from ordinary linear regression.

We fitted separate censored normal models for change in systolic and diastolic BP. We termed models that were adjusted only for the confounders listed above “minimally adjusted”, and models further controlled for the suspected mediators “fully adjusted”. To account for the sex-specific nonlinear dependency of BP on age, we evaluated a set of nine candidate models with different age and sex adjustments using the Akaike Information Criterion (AIC). The best-fitting model contained linear, quadratic and cubic terms of age, each interacting with sex. No other model selection was performed. Effect modification by sex was assessed by introducing the appropriate interaction term. We did not adjust our models for baseline BP, to guard against bias due to the potential fluctuations of measurements^41^. Effect estimates from otherwise identical models with baseline adjustment are reported in the Supplementary Information.

#### Depression and incident hypertension diagnosis

The prospective association between baseline depression and incident hypertension diagnosis was assessed with a logistic regression model. We included the same set of covariates as the fully adjusted models for change in BP, and additionally the baseline systolic and diastolic BP, and the follow-up duration of the wave. Effect modification by sex was assessed by introducing the appropriate interaction term.

All analyses used clustered robust standard errors to account for repeat measurements of participants. Analyses were performed with Stata statistical software, release 16.

## Results

### Participant characteristics

Table 1 displays the baseline participant characteristics by wave. A total of 3214 observations were included in our analyses. About 11% of participants at SAPALDIA2 and 14% at SAPALDIA3 had depression. Depression was slightly less frequent among those who met our inclusion criteria compared to those who did not (12.4% at wave 2 and 15.3% at wave 3). An age-related increase in systolic BP was observed from SAPALDIA2 and SAPALDIA3. There was 12% and 10% incident hypertension diagnosis in wave 2 and wave 3 respectively.

**Table 1:** Baseline^a^ participant characteristics (total n = 3214) by wave for the analysis of the prospective association between depression and change in blood pressure as well as incident hypertension diagnosis.

	Wave 2 (SAPALDIA2 to SAPALDIA3) (n = 2467)	Depressed at wave 2 (criteria iv) (n = 262)	Wave 3 (SAPALDIA3 to SAPALDIA4 55+) (n = 747)	Depressed at wave 3 (criteria iv) (n = 101)
**Depression, frequency (%)**				
i. Depression diagnosis	113 (4.6)		73 (9.8)	
ii. Depressive symptoms (SF-36 MH < 50)	149 (6.0)		31 (4.2)	
iii. History of antidepressant use	83 (3.4)		38 (5.1)	
iv. Presence of depression^b^ (any of i, ii, or ii)	262 (10.6)		101 (13.5)	
Age, mean (SD)	48.0 (10.5)	49.1 (11.0)	58.7 (7.4)	58.5 (7.3)
**Sex, frequency (%)**				
Male	1103 (44.7)	85 (32.4)	311 (41.6)	30 (29.7)
Female	1364 (55.3)	177 (67.6)	436 (58.4)	71 (70.3)
**Education level, freq (%)**				
Primary school	87 (3.5)	21 (8.0)	20 (2.7)	7 (6.9)
Secondary school	1556 (63.1)	165 (63.0)	456 (61.0)	61 (60.4)
Technical College or University	824 (33.4)	76 (29.0)	271 (36.3)	33 (32.7)
**Employment, frequency (%)**				
Employed	1968 (79.8)	187 (71.4)	501 (67.1)	60 (59.4)
House person	302 (12.2)	45 (17.2)	52 (7.0)	11 (10.9)
In training/military service	31 (1.3)	4 (1.5)	5 (0.7)	0 (0)
Not working	25 (1.0)	13 (5.0)	5 (0.7)	3 (3.0)
Pensioner	141 (5.7)	13 (5.0)	184 (24.6)	27 (26.7)
**Area, frequency (%)**				
Basel	299 (12.1)	25 (9.5)	93 (12.5)	10 (9.9)
Wald	494 (20.0)	33 (12.6)	105 (14.1)	12 (11.9)
Davos	196 (7.9)	13 (5.0)	80 (10.7)	6 (6.0)
Lugano	265 (10.7)	29 (11.1)	106 (14.2)	11 (10.9)
Montana	287 (11.6)	47 (17.9)	79 (10.6)	21 (20.8)
Payerne	289 (11.7)	43 (16.4)	96 (12.9)	17 (16.8)
Aarau	406 (16.5)	38 (14.5)	103 (13.8)	10 (9.9)
Geneva	231 (9.4)	34 (13.0)	85 (11.4)	14 (13.9)
Swiss Socioeconomic Position, mean (SD)	64.4 (9.6)	62.5 (9.8)	65.0 (9.2)	63.5 (9.4)
**Smoking status, frequency (%)**				
Never	1211 (49.1)	115 (43.9)	387 (51.8)	50 (49.5)
Former	660 (26.8)	59 (22.5)	235 (31.5)	31 (30.7)
Smoker	596 (24.2)	88 (33.6)	125 (16.7)	20 (19.8)
**Physical activity, frequency (%)**				
Insufficiently active^c^	615 (24.9)	76 (29.0)	151 (20.2)	26 (25.7)
Sufficiently active^d^	1852 (75.1)	186 (71.0)	596 (79.8)	75 (74.3)
**Vegetable consumption, frequency (%)**				
Not daily	1913 (77.5)	196 (74.8)	552 (73.9)	73 (72.3)
Daily	554 (22.5)	66 (25.2)	195 (26.1)	28 (27.7)
**Fruit consumption, frequency (%)**				
Not daily	2212 (85.6)	224 (85.5)	638 (85.4)	73 (72.3)
Daily	355 (14.4)	38 (14.5)	109 (14.6)	28 (27.7)
**Alcohol**				
Less than several times a week	1587 (64.3)	168 (64.1)	444 (59.4)	69 (68.3)
Several times per week	880 (35.7)	94 (35.9)	303 (40.6)	32 (31.7)
Sleepiness^e^, mean (SD)	1.8 (0.4)	1.8 (0.5)	1.8 (0.5)	1.8 (0.5)
Body mass index (kg/m^2^), mean (SD)	24.6 (3.6)	24.4 (3.8)	24.6 (3.7)	24.6 (4.0)
Pulse (beats per minute), mean (SD)	69.4 (9.8)	69.5 (10.3)	67.8 (9.5)	69.9 (10.0)
Systolic BP (mmHg), mean (SD)	116.6 (12.4)	115.0 (12.5)	121.5 (10.2)	119.6 (9.4)
Diastolic BP (mmHg), mean (SD)	74.8 (7.7)	73.4 (7.9)	74.2 (7.1)	74.2 (6.0)
Incident hypertension diagnosis	286 (11.6)	46 (17.6)	71 (9.5)	12 (11.9)

^a^First time point of each wave is defined as baseline.

^b^The primary exposure for this study.

^c^Insufficiently active (< 150 min of moderate physical activity and < 75 min of vigorous physical activity per week).

^d^Sufficiently active (> 150 min of moderate physical activity or > 75 min of vigorous physical activity per week).

^e^Sleepiness—mean score per item of the Epworth Sleepiness Scale.

### Association between depression and change in blood pressure

Table 2 shows the coefficients of the censored regression models for the association between depression and change in systolic and diastolic BP among people who were normotensive at baseline. Depression was also disaggregated first by antidepressant use, then further by antidepressant class. We found that non-disaggregated depression was associated with age-related systolic BP increase both in our minimally adjusted model (β = − 1.99, 95% CI − 4.02 to 0.04) and the model adjusted further for suspected mediators (β = − 2.08, 95% CI − 4.09 to − 0.07), although the former result did not meet the conventional statistical significance threshold of *p* < 0.05. The direction of the association with diastolic BP was the same, but did not reach statistical significance in either model: β = − 0.82, 95% CI − 2.10 to 0.45; and β = − 0.88, 95% CI − 2.15 to 0.39, respectively.

**Table 2:** Prospective association between baseline^a^ depression (binary, and disaggregated by antidepressant use and antidepressant class) and age-related increase in systolic and diastolic blood pressure over 10 years among normotensives at baseline ^a^ (n = 3214).

	Change in systolic blood pressure over 10 years				Change in diastolic blood pressure over 10 years			
	Minimally adjusted ^b^		Fully Adjusted ^c^		Minimally adjusted ^b^		Fully Adjusted ^c^	
	Coef	95% CI	Coef	95% CI	Coef	95% CI	Coef	95% CI
**Presence of depression**								
Not depressed (n = 2851)	(Reference)		(Reference)		(Reference)		(Reference)	
Depressed (n = 363)	− 1.99	(− 4.02, 0.04)	− 2.08	(− 4.09, − 0.07)	− 0.82	(− 2.10, 0.45)	− 0.88	(− 2.15, 0.39)
**Depression, disaggregated by antidepressant medication use**								
Not depressed (n = 2851)	(Reference)		(Reference)		(Reference)		(Reference)	
Depressed, not medicated (n = 242)	− 1.59	(− 4.03, 0.85)	− 1.59	(− 4.01, 0.83)	− 0.58	(− 2.15, 0.99)	− 0.55	(− 2.12, 1.01)
Depressed, medicated (n = 121)	− 2.80	(− 6.15, 0.56)	− 3.03	(− 6.35, 0.29)	− 1.30	(− 3.31, 0.72)	− 1.52	(− 3.50, 0.46)
**Depression, disaggregated by type of antidepressant**								
Not depressed (n = 2851)	(Reference)		(Reference)		(Reference)		(Reference)	
Depression, not medicated (n = 242)	− 1.60	(− 4.04, 0.83)	− 1.60	(− 4.02, 0.82)	− 0.59	(− 2.16, 0.98)	− 0.56	(− 2.12, 1.01)
Depressed, on N06AA^d^ (n = 16)	− 5.75	(− 12.58, 1.08)	− 5.70	(− 12.22, 0.82)	− 5.27	(− 9.74, − 0.81)	− 5.47	(− 9.61, − 1.33)
Depressed, on N06AB^e^ (n = 55)	− 4.24	(− 9.00, 0.52)	− 4.39	(− 9.16, 0.37)	− 1.17	(− 4.02, 1.67)	− 1.12	(− 3.92, 1.67)
Depressed, on other or multiple antidepressants (n = 46)	0.09	(− 5.67, 5.85)	− 0.35	(− 6.01, 5.31)	− 0.04	(− 3.57, 3.49)	− 0.63	(− 4.10, 2.84)

^a^First time point of each wave is defined as baseline.

^b^Censored normal regression models “minimally adjusted” included age, quadratic age term, cubic age term, sex, age and sex interactions, education, employment, SSEP, study area, and wave.

^c^Fully adjusted models included all of the above and BMI, pulse, sleepiness, physical activity, fruit consumption, vegetable consumption, alcohol, and smoking.

^d^N06AA—Non-selective monoamine reuptake inhibitors.

^e^N06AB—Selective serotonin reuptake inhibitor.

### Effect modification and mediation of the association of depression with change in blood pressure

We found no evidence that the association of depression with age-related increase in systolic or diastolic BP differed by sex (P_interaction_ = 0.45 and 0.54, respectively), nor by antidepressant use (P_interaction_ = 0.48 and 0.44, respectively).

The direction of effect modification with antidepressant use and antidepressant classes may be of biological interest and is therefore presented as input for future, larger studies. The association of depression with lower age-related increase in systolic BP tended to be weaker in persons without a history of antidepressant use (β = − 1.59, 95% CI − 4.01 to 0.83), but stronger among persons with a history of antidepressant use (β = -3.03, 95% CI − 6.35 to 0.29) in the fully adjusted models,. Similar but also statistically non-significant patterns were observed for diastolic BP where associations weakened in depressed persons without antidepressant use (β = − 0.55, 95% CI − 2.12 to 1.01) and were stronger in the case of antidepressant use (β = − 1.52, 95% CI − 3.50 to 0.46).

We also observed suggestive differences between antidepressant classes, although the confidence intervals of all findings also crossed zero. Depressed persons using non-selective monoamine reuptake inhibitors (NO6AA) had the largest lowering in age-related increase of systolic BP (β = − 5.70, 95% CI − 12.22 to 0.82) among antidepressant classes, although age-related increase in systolic BP was also lowered in all other classes but with even less confidence. Equivalent patterns were observed for diastolic BP.

We found that controlling the minimally adjusted model further for covariates related to lifestyle (smoking, physical activity, alcohol, sleepiness, fruit consumption, vegetable consumption) and autonomic nervous system (pulse) had little impact on the estimates of the effect of depression, which is consistent with no or little mediation by these factors).

### Association between depression and incident hypertension diagnosis

Table 3 shows the odds ratios of incident hypertension diagnosis at follow-up. There were a total of 286 events of hypertension diagnosis (11.6%) in wave 2, and 71 events (9.5%) in wave 3. There was a clear increase in odds for depressed persons to receive a diagnosis of hypertension at follow-up (OR 1.86, 95% CI 1.33 to 2.60). This increase was larger and statistically significant for the participants without a history of antidepressant use (OR 2.23, 95% CI 1.53 to 3.27), but weaker and not statistically significant for those with antidepressant use (OR 1.21, 95% CI 0.66 to 2.24). We found no evidence that the effect of depression on incident hypertension diagnosis differs by sex (P_interaction_ = 0.77).

**Table 3:** Prospective association between baseline^a^ depression (binary, disaggregated by antidepressant use, and disaggregated by antidepressant class) and incident hypertension diagnosis (self-reported physician diagnosis or antihypertensive treatment use at follow-up) among normotensives at baseline^a^ (n = 3214).

	Incident hypertension diagnosis	
	Odds ratio	95% CI
**Depression**		
No depression (n = 2851)	(Reference)	
Depressed (n = 363)	1.86	(1.33, 2.60)
**Depression**		
No depression (n = 2851)	(Reference)	
Depression, not medicated (n = 242)	2.23	(1.53, 3.27)
Depression, medicated (n = 121)	1.21	(0.66, 2.24)
**Depression**		
No depression (n = 2851)	(Reference)	
Depression, not medicated (n = 242)	2.22	(1.52, 3.26)
Depressed, on N06AA^b^ (n = 16)	0.55	(0.06, 4.93)
Depressed, on N06AB^c^ (n = 55)	0.85	(0.32, 2.25)
Depressed, on other or multiple antidepressants (n = 46)	2.02	(0.88, 4.64)

A total of 357 incidences of hypertension diagnosis (11.1%) were observed. Logistic regression models with adjustment for age, sex, education, employment, Swiss SEP, area, wave, BMI, pulse, daytime sleepiness, physical activity, fruit consumption, vegetable consumption, alcohol, smoking, baseline systolic and diastolic BP, and years of follow-up.

^a^First time point of each wave is defined as baseline.

^b^N06AA—Non-selective monoamine reuptake inhibitors.

^c^N06AB—Selective serotonin reuptake inhibitor.

## Discussion

We found that in normotensive people, the age-related increase in systolic BP was lower by about 2 mmHg among depressed compared to non-depressed participants over 10 years of follow-up. However the presence of depression at baseline increased the odds of having hypertension diagnosed if it developed.

### Predictive association between baseline depression and change in blood pressure

#### Main effect

To the best of our knowledge, our study is the first to report the predictive association between depression and change in BP as a continuous variable among normotensive people and incident hypertension diagnosis in the same study sample. While BP tends to increase with age, we found that systolic BP increased less among people with depression compared to those without depression at baseline. This association did not vary by sex in our study, although there is evidence of effect modification by sex in another study^31^ which included people with hypertension. Our observations suggest that systolic BP is not a relevant mediator in the causal effect of depression on CVD as it was recently confirmed in the context of a bi-directional Mendelian Randomization (MR) study^42^. In the MR study, a slight attenuation of the causal effect of depression on CVD was observed after adjustment for BP, but neither was a mediation analysis conducted, nor was the sample restricted to normotensive persons.

Few other longitudinal studies have assessed depression and BP as a continuous variable. Our findings are consistent with one general population-based study (age 40 ± 10.6) which included both normotensive and hypertensive people and found that a higher depression symptoms score (continuous) was predictive of attenuated BP after 11 years of follow-up^29^, as was depression as a binary exposure, albeit with less confidence. A second study, which was restricted to persons with hypertension, did not find that depression at case-level was predictive of change in BP (β = − 1.3, 95% CI − 5.3 to 2.7)^30^); however when they stratified depression by severity, they found that moderate major depressive disorder (but not mild or severe) predicted lower BP (systolic BP (β = − 7.5, 95% CI − 13.2 to − 1.9) and diastolic BP (β = − 4.5, 95% CI − 7.8 to − 1.3))^30^. Studies with contrasting evidence to our findings did not assess BP change over time^31^ or were restricted to women^17^.

There are two main hypotheses which need to be discussed in the context of our finding that depression attenuates age-related BP increase. Firstly, it has been hypothesized before that lower BP among depressed persons may be due to a higher use of antihypertensive drugs^14^. Our analyses provide no support for this hypothesis, since the effect was observed even though we removed baseline antihypertensive drug users from the sample and controlled for initiation mid-wave with censored regression. However, as our medication data is self-reported, the possibility of differential misclassification remains. The second hypothesis argues for a shared biology between depression and low BP, and therefore not a causal pathway between the two. The monoamine theory of depression^43^ suggests that the underlying pathogenesis of depression is a depletion in the levels of serotonin, norepinephrine, and/or dopamine in the central nervous system. Monoamines also play a central role in raising BP and therefore low BP is observed when there is a depletion of these monoamines. Thus when there is a depletion of monoamines, both depressive symptoms and low BP could be observed.

The effect size of the lower age-related increase in systolic BP (depressed people had about 2 mmHg smaller increase in systolic BP than non-depressed over 10 years) may not be of clinical significance at the individual level. However at the level of the population it will decrease substantially the number of individuals becoming hypertensive due to the slight shift in the distribution of BP towards lower values. This preventive effect is not irrelevant given the high prevalence of both depression and hypertension. Furthermore, our findings have important implications on cardiovascular epidemiology. Hypertension as a potential mechanism linking depression and CVD is in the literature spotlight. However, our findings suggest that the association between depression and CVD is not mediated through hypertension. Therefore epidemiological studies investigating other potential mechanisms are warranted.

#### Effect modification antidepressant use

In addition to assessing depression as a binary exposure, we also stratified depression by antidepressant use. When compared to persons without depression, we observed that participants reporting depression with a history of antidepressant treatment may have a greater attenuation in systolic and diastolic BP change than did participants reporting depression without a history of antidepressant treatment. This statistically non-significant finding was unexpected in light of the fact that antidepressants increase monoamines in the brain to improve depressive symptoms. Also other researchers did not find effect modification by general antidepressant use in longitudinal studies, however they were not restricted to normotensive persons^29,30^. We further conducted an analysis disaggregating depression by antidepressant class and found suggestive, but not statistically significant, evidence that attenuation of BP course may be strongest for N06AA class of antidepressants. This is in contrast to one cross-sectional study which found that certain subclasses of antidepressants (tricyclic antidepressants as well as noradrenergic and serotonergic acting antidepressants) increased BP^14^. In addition, a meta-analysis of Randomized Controlled Trials found that although the N06AB class of antidepressants did not change BP, N06AA led to a modest increase in systolic and diastolic BP when compared to N06AB^44^. The reporting of these suggestive results arising from a small study sample is meant to stimulate further investigation in the context of larger cohorts.

There are two potentially opposite effects of antidepressants to be considered to explain the trends in our findings. The first is the short-term action, whereby the antidepressant increases monoamines in the brain and therefore neurotransmitters which are responsible for signaling vasoconstriction and increased BP. The second is in the long term action consistent with our findings. Antidepressant therapy aims to alleviate depressive symptoms, thereby potentially removing the effect of depression on BP attenuation. A history of antidepressant use may however be a proxy for longer exposure and/or more severe depression, given that there are clearer benefits of antidepressant treatment for more severe and longstanding depression^45^. Given that the N06AB antidepressant class is currently the recommended first-line antidepressant treatment, the apparent strongest effect in N06AA users observed in our study might be an indication of longer-standing and/or treatment-resistant depression.

#### Effect mediation

We saw no meaningful attenuation in any effect estimate between the corresponding minimally and fully adjusted models (Table 2). This might indicate weak or no mediation by the factors that we considered. Depression however is a complex disease that has different clinical presentations, for example increase versus decrease in appetite and sleep, masking the mediating effect of such factors in single depression category analyses. For this reason, some studies differentiate subtypes of depression^46,47^, which could not be assessed in the present study. The biggest limitation of our approach to mediation was that depression and the hypothesized mediators were measured at the same time. Future studies seeking to elucidate the mechanisms linking depression and BP should ideally be based on longitudinal designs with more frequent follow-ups.

### Predictive association between baseline depression and incident hypertension diagnosis

Baseline depression was associated with higher odds of incident hypertension diagnosis at follow-up compared to no depression. Our findings are consistent with the large body of evidence on the association between depression and the incidence of hypertension, which often includes hypertension diagnosis solely or as part of its definition^15,16,18,48,49^. We interpret our findings as an indication that people with depression are more likely to have underlying hypertension diagnosed, likely mediated by increased healthcare-seeking behaviour^28^. We could not adjust for healthcare utilization in our study because data collection on health-seeking behaviours began in SAPALDIA4.

Our analyses indicate that depression increases the likelihood of being diagnosed and treated for hypertension through mechanisms that might not involve increasing BP. This finding has an important methodological implication for future studies of the depression-BP relationship. Because depression and increased BP modify the likelihood of hypertension treatment independently, adjusting analyses for treatment introduces collider bias^50^. The effects of depression and antihypertensive medication can be disentangled more easily in longitudinal studies with multiple and more frequent follow-ups.

### Strengths and limitations

This study points to the importance of disentangling the mixed evidence on the predictive association between depression and the course of BP by assessing in the same sample of normotensive persons the prospective association of depression with BP course and with obtaining a new diagnosis of hypertension. The longitudinal design allowed for a temporal sequence of exposure and outcome. The inclusion of SF-36 MH score to identify potential cases of undiagnosed depression minimized exposure misclassification in our study and allowed for identifying persons with depressive symptoms in the absence of treatment. The association remained when we removed the SF-36 MH criteria from the exposure definition in a sensitivity analysis. The association between diagnosed depression and age-related increase in systolic BP in the fully adjusted model was − 2.65 (95-CI − 4.95 to − 0.35) and − 1.54 (95 CI − 2.86 to − 0.21) for diastolic BP.

One limitation of our study was that we did not measure the duration of exposure and severity of depressive symptoms. Observing a dose–response effect would increase the evidence for a causal relationship for shared biological pathways, as was done in another study^29^. We did not consider persistent depression in order to maintain the prediction perspective and to avoid reverse causation bias in the light of only three follow-up time points. An important limitation of the study is the fact that BP was only measured at three time points several years apart. Therefore, the calculated changes in BP between these points may incorporate intra-individual fluctuations of BP over short time intervals. A limitation of any cohort is the loss of participants to follow-up. In light of the restriction of the study sample to normotensive persons, it is very difficult to judge bias due to loss of follow-up. We observed that depression was slightly less prevalent in the study sample. We may have underestimated the effect of depression in the course of BP in case participants lost to follow-up in this cohort had more depression and at the same time more hypertension.

The relatively small sample size may be a limitation to our study. However, only one similar study^29^ had a larger sample size (n = 17 410), while others were significantly smaller (n < 2100)^17,20,30,31^. The results of this study can guide research approaches in mega-cohorts established more recently and currently being followed up in various countries.

## Conclusion

In normotensive participants without a history of hypertension or antihypertensive treatment, depression goes along with an attenuation of the age-related increase in systolic BP, possibly rooted in a central monoamine deficiency underlying both depression and low BP. The effect is unlikely to be clinically relevant at the level of the individual, but shifts the distribution of BP towards lower values at the population level. At the same time, the presence of depression or depression symptoms at baseline was predictive of a higher likelihood for obtaining a hypertension diagnosis during follow-up, possibly the result of increased healthcare-seeking behaviour among depressed people. Further disentangling the inconsistencies in the literature and understanding the pathways from depression to high BP, hypertension and CVD is of public health relevance given the contribution of these phenotypes to disease burden worldwide.

## Supplementary Information

Supplementary Table S1.

## Data Availability

The datasets analysed during the current study are not publicly available due to the protection of non-anonymized data in the context of cohort data, but are available from the corresponding author on reasonable request.
