# Supplementary material for: Depression and cardiovascular disease are not linked by high blood pressure: findings from the SAPALDIA cohort
Source: Sci Rep. 2022 Apr 1;12:5516. doi: 10.1038/s41598-022-09396-2 (PMC8975826; doi:10.1038/s41598-022-09396-2)
Supplement: Supplementary file 1 — Supplementary Table S1. [file 41598_2022_9396_MOESM1_ESM.docx]

**Supplementary Information to Article “**Depression and cardiovascular disease are not linked by high blood pressure: Findings from the SAPALDIA cohort**”**

**Table S1**. Prospective association between baseline ^a^ depression (binary, disaggregated by antidepressant use and antidepressant class) and age-related increase in systolic and diastolic blood pressure over 10 years among normotensives at baseline a (n=3214), with adjustment for baseline blood pressure.

|  | **Change in systolic blood pressure over 10 years** | | | | **Change in diastolic blood pressure over 10 years** | | | |
| --- | --- | --- | --- | --- | --- | --- | --- | --- |
|  | Lightly adjusted ^b^ | | Fully Adjusted ^c^ | | Lightly adjusted ^b^ | | Fully Adjusted ^c^ | |
|  | Coeff. | 95% CI | Coeff. | 95% CI | Coeff. | 95% CI | Coeff. | 95% CI |
| Presence of depression  Not depressed (n=2851)  Depressed (n=363) | (reference)  -2.22 | (-4.20, -0.23) | (reference)  -2.30 | (-4.26, -0.35) | (reference)  -0.98 | (-2.19, 0.24) | (reference)  -1.05 | (-2.23, 0.14) |
| Depression, disaggregated by antidepressant medication use  Not depressed (n=2851)  Depressed, not medicated (n=242)  Depressed, medicated (n=121) | (reference)  -1.77  -3.10 | (-4.15, 0.60)  (-6.38, 0.19) | (reference)  -1.69  -3.50 | (-4.04, 0.65)  (-6.73, -0.26) | (reference)  -0.69  -1.55 | (-2.18, 0.80)  (-3.45, 0.35) | (reference)  -0.59  -1.94 | (-2.06, 0.87)  (-3.76, -0.11) |
| Depression, disaggregated by type of antidepressant  Not depressed (n=2851)  Depression, not medicated (n=242)  Depressed, on N06AA ^d^ (n=16)  Depressed, on N06AB ^e^ (n=55)  Depressed, on other or multiple antidepressants (n=46) | (reference)  -1.79  -6.66  -4.59  0.07 | (-4.16, 0.59)  (-13.27, -0.06)  (-9.19, 0.01)  (-5.66, 5.79) | (reference)  -1.71  -6.67  -4.88  -0.62 | (-4.05, 0.63)  (-12.92, -0.41)  (-9.46, -0.29)  (-6.22, 4.98) | (reference)  -0.70  -5.37  -1.70  0.02 | (-2.18, 0.79)  (-9.74, -1.00)  (-4.45, 1.05)  (-3.18, 3.22) | (reference)  -0.60  -5.48  -1.85  -0.79 | (-2.07, 0.87)  (-9.57, -1.39)  (-4.47, 0.78)  (-3.90, 2.32) |

*^a^First time point of each wave is defined as baseline, ^b^ Censored normal regression models “lightly adjusted” included baseline blood pressure, age, quadratic age term, cubic age term, sex, age and sex interactions, education, employment, SSEP, study area, and wave.* ^c^ *Fully adjusted models included all of the above and BMI, pulse, sleepiness, physical activity, fruit consumption, vegetable consumption, alcohol, and smoking.* ^d^ *N06AA –Non-selective monoamine reuptake inhibitors;* ^e^ *N06AB - Selective serotonin reuptake inhibitor.*
